# Maximum Surgical Blood Ordering Schedule in Common Pediatric Cardiac Surgeries in a Tertiary Center

**DOI:** 10.30699/IJP.2023.545972.2804

**Published:** 2023-10-15

**Authors:** Maryam Sotoudeh Anvari, Seyedeh Zohreh Hashemi, Mohammadreza Mirzaaghayan, Alireza Abdollahi, Mohammad Taghi Haghi Ashtiani, Abbas Akbari

**Affiliations:** 1 *Department of Surgical and Clinical Pathology, Children Medical Center, Tehran University of Medical Sciences, Tehran, Iran *; 2 *Tehran University of Medical Sciences, Tehran, Iran*; 3 *Department of Cardiac Surgery, Children Medical Center, Tehran University of Medical Sciences, Tehran, Iran*; 4 *Department of Pathology, Imam Hospital Complex, School of Medicine, Tehran University of Medical Sciences, Tehran, Iran *

**Keywords:** Blood Transfusion, Cardiac Surgery, Pediatrics

## Abstract

**Background & Objective:**

Unnecessary pre-operative ordering of red blood cells (RBCs) in elective surgeries increases costs and waste of blood inventory. Maximum surgical blood order schedule (MSBOS) is a helpful strategy in the estimation of blood units needed for surgery and the prevention of overconsumption. In this study, an MSBOS for pediatric cardiac surgeries is designed.

**Methods:**

In this cross-sectional study, we included all pediatric patients who underwent elective cardiac surgery in Children’s Medical Center in Tehran, Iran, from March 21, 2019, to September 22, 2019. Data consisted of the type of surgery and the number of blood units transfused and units cross-matched, based on which cross-match to transfusion ratio (CTR), the transfusion index (TI), and transfusion probability (T%) were calculated.

**Results:**

Overall 205 pediatric patients were included in the study. Four hundred and ten RBCs units were cross-matched, and 262 were transfused. The overall results of the CTR, T%, and TI for all the eight types of cardiac surgery were 1.56 (410/262), 76% (157/205), and 1.28 (262/205), respectively. The raw MSBOS for cardiac surgeries included ventricular septal defect, tetralogy of fallot, dextro-transposition of the great arteries, atrial septal defect, aortic coarctation, patent ductus arteriosus, pulmonary stenosis, and pacemaker insertion, which were 1.58, 1.03, 1.54, 1.66, 0.77, 0, 1.25, and 0 unit, respectively, and the figures were rounded up.

**Conclusion:**

Accurate MSBOS protocols reduce cross-match workload in laboratories, lead to the appropriate use of blood stocks with less wastage, save human and economic resources, and eventually, promote patient safety.

## Introduction

Blood transfusions plays a significant role in the safe supply of blood products and the management of surgical patients. Several strategies have been developed as a common practice in elective and emergency surgical procedures (1).

Excessive blood ordering and the lack of a particular schedule for requisition of blood products are among the most common problems in teaching hospitals, leading to increased costs and waste of limited blood resources (1-3). 

In the developing countries, about 40-70% of the requested blood units are used in elective surgeries, and only 64% of the cross-matched blood units are transfused (4-6). Studies show that 31% of blood units are utilized in cardiac and orthopedic surgeries. In thoracic surgeries, bleeding varies from 1% to 3.7%, and the blood transfusion rate ranges from 20% to 52% (7-9).

Among pediatric patients, the highest rate of allogeneic red blood cells (RBCs) preparation, was reported in cardiac surgery with cardiopulmonary bypass (CPB), more specifically, 79% of all procedures involved transfusion (10). 

Variations in transfusion rate could be due to differences in the predefined threshold level of hemoglobin, below which the patient needs blood transfusion, various surgical and anesthetic techniques, cancellation of cases, differences in case-mix, preoperative anemia, and lack of transfusion protocols (11).

In 1975, cross-matched to blood transfusion (C / T) ratio was initiated and widely used to monitor the effectiveness of blood utilization. Ideally, this ratio should be 1.0, but a ratio of 2.5 or below is recommended as an indicator of efficient blood usage transfusion index (TI), and transfusion probability (T%), as described by Mead *et al.* in 1980, is indicative of significant blood usage. A value of 0.5 or higher for TI and a value of 30% and above for T% have been suggested as appropriate blood usage (12, 13). Non-usage probability (NUP) and wastage as a percentage of an issue (WAPI) are two newer indicators defined to improve blood consumption (14, 15).

Non-use probability (NUP) is the total units not transfused/total units requested. High NUP indicating inefficient blood usage, and NUP decreases as the blood-ordering practice improve (15). 

Wastage as a percentage of an issue is defined as the sum of wasted units for each component /sum of units issued for each component at the hospital.(16).

The four common areas of focus for wastage include: (1) units expired after issue from the blood bank but before transfusion not due to reasons other than storage temperature compliance; (2) units wasted due to improper transport or storage; (3) non-stored units returned more than 30 minutes from issue; (4) other waste causes namely breakage, contamination, and pneumatic tube system failure (14). 

Patient blood management (PBM) is a set of multidisciplinary approaches that enhance patients’ medical and surgical outcomes through better clinical management (17-19).

The Maximum Surgical Blood Order Schedule (MSBOS), as part of PBM, is a list of hospital-based, elective surgical procedures to estimate the number of blood product units required to reduce the demand of 90% of patients pre-operatively. It is essentially a table consisting of two columns: 1) surgery name and 2) the number of units crossmatched /type and screen (T&S)/ and no testing required. In these tables, plasma products and platelets concentrate are not included. The maximum surgical blood order schedule reduces unnecessary compatibility testing, prevents the outdating of blood units, saves time, and money, and ultimately, enhances the quality of care (11, 20-22). 

The published MSBOS guidelines are limited in pediatric surgery. Blood products management in pediatric surgeries is required to minimize the risk of resources shortage. Studies show that while the number of blood transfusions increases annually, the number of donors has either remained constant or diminished (23-25). 

The calculation of the mentioned indices related to pediatric surgeries is less discussed in the literature. Therefore, in this study we aimed to compile an MSBOS table for common pediatric cardiac surgeries in a tertiary center to provide guidelines.

## Material and Methods

In this retrospective cross-sectional study conducted from March 21 to September 22, 2019, we assessed pediatrics (≤ 16 years old) who underwent cardiac surgery mainly for congenital heart diseases in the Division of Pediatric Cardiac Surgery in the Children’s Medical Center, Tehran, Iran. Blood utilization was investigated using the cross-matched to transfusion ratio (CTR), transfusion index (TI), transfusion probability (T%), and MSBOS. These indices were calculated based on the following formulas (26):

Number of unit's cross-matched

1.
$\mathrm{CTR}=\frac{\mathrm{Number of unit's cross}-\mathrm{matched}}{\mathrm{Number of units transfused}}$

1.
$\mathrm{T\%}=\frac{\mathrm{Number of patients transfused}\times 100}{\mathrm{Number of patients cross}-\mathrm{matched}}$

1.
TI=Number of units transfused Total number of patients cross-matched

Total number of patients cross-matched.

In our hospital, the MSBOS was defined based on TI, not 1.5 x TI used in some articles because we used adult blood bags (average 250 mL) for all children as pediatric bags were not present in the blood bank at the time (26).

Cross-matched to transfusion ratio is a measure of blood ordering efficiency with a CTR value of greater than 2 indicating significant unutilized blood. T% higher than 30 shows an essential need for blood during surgery. TI refers to the average number of units in a specific surgical procedure. For TI, a value of 0.5 or over is considered meaningful blood utilization.

Finally, MSBOS was calculated based on average blood consumption per surgery. All the stages of this research were performed under the supervision and approval of the Research Ethics Committee of Tehran University of Medical Sciences.

## Results

From March 21 to September 22, 2019, 205 pediatrics who underwent cardiac surgeries were examined in the Children’s Medical Center in Tehran, Iran. The age range was 7 days to 11 years with a weight range of 2200 grams to 25 kilograms. Those with massive transfusion and transfusion reactions were excluded. 

Antibody screening was performed for all the 205 patients. Overall, 157 patients received blood transfusion, 410 cross-matched RBC units were reserved, and 262 units were transfused. Consequently, 76% of units were transfused to 157 patients. 

After categorizing surgeries by cardiac surgeon teams, determining the number of each operation per disease, and calculating the CTR, T%, and TI for each type of surgery (Table 1), the MSBOS was computed (Table 2).

It is worth mentioning cumulative CTR, T% and TI were 1.56 (410/262), 76% (157/205), and 1.28 (262/205), respectively.

**Table 1:** The number and indices for MSBOS calculation

Surgery type	Number of surgery	Number of cross matched patients	Number of transfused patients	Number of cross matched units	Number of transfused units	CTR	T%	TI
VSD	64	64	56	128	101	1.2	0.81	1.58
TOF	29	29	19	58	30	1.93	0.65	1.03
DTGA	23	23	11	26	20	1.3	0.85	1.54
ASD	15	15	15	30	25	1.2	1	1.66
PDA	3	3	0	6	0	0	0	1.25
PS	4	4	4	8	5	1.6	1	0.77
COA	13	13	9	26	10	2.6	0.69	0
Pacing	5	5	0	10	0	0	0	0

VSD: Ventricular septal defect, TOF: Tetralogy of Fallot, DTGA: Dextro-Transposition of the Great Arteries, ASD: Atrial septal defect, COA: Aortic Coarctation, PDA: Patent ductus arteriosus, PS: Pulmonary stenosis, and Pacing: Pacemaker insertion

**Table 2:** Calculated MSBOS for each type of cardiac surgery (sum of number of the patients X number of each unit (3,2,1) divided total number of patients)

Surgery	calculation	Raw MSBOS	Rounded MSBOS	Recommended MSBOS *
VSD	1x3+43x2+12x1+8x0/64	1.58	2	2
TOF	11x2+8x1+10x0/29	1.03	2	2
DTGA	9x2+2x1+2x0/13	1.54	2	2
ASD	10x2+5x1/15	1.66	2	2
PDA	3x0/3	0	0	T&S
PS	1x2+3x1/4	1.25	2	2
COA	1x2+8x1+4x0/13	0.77	1	2
Pacing	5x0/5	0	0	T&S

* The suggested MSBOS values from the Henry's Clinical Diagnosis and Management’s book.

## Discussion

The MSBOS is valuable in optimizing pre-operative transfusion ordering practices (27). Articles with a focus on pediatric patients’ MSBOS, are very limited. In the study by Friedman *et al.* (1979) patients under 18 years of age were excluded from the study due to the variable volumes of pediatric surgeries required (26, 28).

Guzman *et al.* in 2019, conducted a cross-sectional study among patients under 18 years old at the Children's Medical Center of the Philippines. Major abdominal surgery (intestinal atresia, congenital diaphragmatic hernia repair, Ladd's procedure, intussusception, bowel resection, and adhesiolysis), major thoracic surgery (i.e., Lobectomy, esophageal atresia surgery, mediastinal surgery), and hepatobiliary surgery had a C/T ratio of less than 2.0. After MSBOS calculation, one RBCs unit was suggested for each procedure. Although cardiac surgeries were excluded in this study based on the author’s concept of liberal blood transfusion in this type of surgery, CTR, T%, TI, and MSBOS for major thoracic surgery were determined 1.74, 64.4%, 0.69 , and 1.03 , respectively, which are in alignment with our overall results (28). 

We compared our results to strategies for ordering blood for elective surgery outlined in Henry's Clinical Diagnosis and Management’s book as a reference of clinical pathology. These guidelines present the numbers of blood units and associated surgeries at three different institutions. Although this table does not differentiate between pediatrics and the adult population, our data showed high similarity to its content (Table 2).

Mangwana *et al.* studied cardiothoracic surgery blood utilization in a tertiary care hospital, in India. Out of 2752 units cross-matched, 1296 units were transfused, and the C/T ratio was 1.34. Overall, TI and T% were 1.22 and 83.07%, respectively. The table of surgeries included coronary artery bypass graft surgery with, and without valvular surgery, vascular surgery, and thoracic surgery. Although it seems that the study focused on adults, their overall results are in line with our findings (22, 29). 

Recently, focusing on personalized medicine, Feng *et al.* introduced a novel approach using a machine learning algorithm before liver transplantation. They created an artificial intelligence model to predict the appropriate amount of preoperative RBC demand, using several preoperative analysis parameters, such as portal hypertension, age, hemoglobin, diagnosis, direct bilirubin, activated partial thromboplastin time, globulin, aspartate aminotransferase, and alanine aminotransferase (30) 

According to Park *et al.*’s study in South Korea and the MSBOS comparisons conducted regarding 1989, 1999, 2007, and 2013 tables demonstrated a significant declining trend. Therefore updating MSBOS tables in each blood transfusion service can improve blood transfusion management (31).

Our data showed that most elective pediatric cardiac surgical procedures such as VSD, TOF, DTGA, ASD, PDA, and pulmonary stenosis, need cross-matching one to two adult units, while for aortic coarctation and pacing, typing and screening are sufficient.

This study had several limitation such as not having access to some data in our blood bank database, including the risk factors for each patient, preoperative hemoglobin concentration, patient weight, and comorbidities, and using adult RBC units in pediatrics. Future studies can examine the impact of the need for blood transfusion in the development of MSBOS protocol.

## Conclusion

The MSBOS creates a sense of security in the laboratory, saves time, and ultimately creates a good teamwork connection between the operating room and the blood bank, which eventually promotes patient's health. Use of the machine learning algorithms to predict the exact amount of pre-operative blood demand can be an efficient method to reduce blood unit waste; however, further studies are recommended for better management of blood reserves in pediatric surgeries.

## Funding

None.

## Conflict of Interest

There are no conflicts of interest.
